# Clearance of therapy‐induced senescent tumor cells by the senolytic ABT‐263 via interference with BCL‐X_L_–BAX interaction

**DOI:** 10.1002/1878-0261.12761

**Published:** 2020-08-05

**Authors:** Tareq Saleh, Valerie J. Carpenter, Liliya Tyutyunyk‐Massey, Graeme Murray, Joel D. Leverson, Andrew J. Souers, Moureq R. Alotaibi, Anthony C. Faber, Jason Reed, Hisashi Harada, David A. Gewirtz

**Affiliations:** ^1^ Department of Basic Medical Sciences Faculty of Medicine The Hashemite University Zarqa Jordan; ^2^ Departments of Pharmacology & Toxicology School of Medicine Massey Cancer Center Virginia Commonwealth University Richmond VA USA; ^3^ Department of Physics Virginia Commonwealth University Richmond VA USA; ^4^ AbbVie North Chicago IL USA; ^5^ Department of Pharmacology and Toxicology College of Pharmacy King Saud University Riyadh Saudi Arabia; ^6^ Philips Institute for Oral Health Research School of Dentistry Massey Cancer Center Virginia Commonwealth University Richmond VA USA

**Keywords:** ABT‐263, BCL‐X_L_, chemotherapy, radiation, senescence, senolytic

## Abstract

Tumor cells undergo senescence in response to both conventional and targeted cancer therapies. The induction of senescence in response to cancer therapy can contribute to unfavorable patient outcomes, potentially including disease relapse. This possibiliy is supported by our findings that tumor cells induced into senescence by doxorubicin or etoposide can give rise to viable tumors *in vivo*. We further demonstrate sensitivity of these senescent tumor cells to the senolytic ABT‐263 (navitoclax), therefore providing a “two‐hit” approach to eliminate senescent tumor cells that persist after exposure to chemotherapy or radiation. The sequential combination of therapy‐induced senescence and ABT‐263 could shift the response to therapy toward apoptosis by interfering with the interaction between BCL‐X_L_ and BAX. The administration of ABT‐263 after either etoposide or doxorubicin also resulted in marked, prolonged tumor suppression in tumor‐bearing animals. These findings support the premise that senolytic therapy following conventional cancer therapy may improve therapeutic outcomes and delay disease recurrence.

## Graphical abstract

Senescent tumor cells can be selectively eliminated by the BH3 mimetic, ABT‐263 (navitoclax), via senolysis. We show that ABT‐263 inhibits the interaction between anti‐apoptotic BCL‐2 family member, BCL‐X_L_, and pro‐apoptotic effector, BAX. This results in senescent tumor cell death *in vitro* and reduced tumor volume *in vivo*. This work highlights the utilization of senolytic agents to enhance efficacy of anticancer therapy.

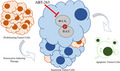

**Abbreviations**
Dox: doxorubicin
Eto: etoposide
HSLCI: high‐speed live cell interferometry
IR: ionizing radiation
SASP: senescence‐associated secretory phenotype
SA‐β‐Gal: senescence‐associated beta galactosidase
TIS: therapy‐induced senescence

## 1 Introduction

Senescence is a cellular state that manifests as a stable cell cycle arrest [1]. The senescent phenotype encompasses a spectrum of features including macromolecular damage, altered genetic and epigenetic expression, dysregulated metabolism, apoptosis resistance, and a secretome comprised of various inflammatory mediators, growth factors, and enzymes collectively termed the senescence‐associated secretory phenotype (SASP) [2, 3]. Senescence contributes to tumor suppression by preventing the replication of cells at risk of malignant transformation [4]. Consequently, senescent cells accumulate in both premalignant and malignant lesions [5]. Senescence is also induced in tumor cells by conventional and targeted chemotherapy and radiotherapy, making senescence a primary response to cancer treatment [6].

Although senescence has been conventionally defined as an irreversible form of growth arrest, there is ample evidence that cells expressing hallmarks of senescence can re‐emerge and enter a proliferative state (reviewed in Refs [6, 7]). For instance, cells evade replicative senescence (RS) when p53 function is suppressed [8], and can escape from oncogene‐induced senescence when telomerase reverse transcriptase (hTERT) gene expression is de‐repressed [9]. We have recently reported the escape/proliferative recovery of tumor cells (lung, breast, and colon) from therapy‐induced senescence (TIS), and that senescent tumor cells can give rise to proliferating progeny capable of tumor formation upon implantation in mice [10, 11]. These findings indicate that senescent tumor cells may enter a temporary—rather than permanent—growth arrest, from which these cells can escape and potentially contribute to disease recurrence [12]. This premise is supported by the recent evidence, suggesting that a subpopulation of senescent cells is capable of eluding immunosurveillance through secretory pathways [13, 14]. In fact, accumulating senescent cells are strongly linked to several adverse outcomes of conventional genotoxic therapy through the promotion of inflammatory responses [15]. As such, the persistence of senescent tumor cells after treatment is likely to represent a suboptimal and ultimately undesirable therapeutic response [12].

In parallel, recent efforts have shown that the selective removal of senescent cells from progeroid animals delays the onset of several aging phenotypes and ameliorates established pathologies [16]. This approach (based on the p16^INK4^ suicide gene INK‐ATTAC model) has been extended to identifying novel small molecules (i.e., *senolytics*) that can selectively eliminate senescent cells [17, 18, 19]. Initial efforts showed that senolytics were successful in mitigating a plethora of aging‐associated pathologies [20, 21, 22]. Moreover, senolytics have successfully improved the outcome of other disorders such as myocardial infarction, obesity, and diabetes mellitus, where accumulating senescent cells have a clear pathogenetic role [23, 24]. In this context, a powerful model to eliminate senescent cells is the use of the established BCL‐2/BCL‐X_L_ inhibitor, ABT‐263 (navitoclax) [25] as a senolytic adjuvant in cancer therapy [26]. While ABT‐263 is usually applied in combination with chemotherapy, we have tested a novel sequential therapeutic approach; specifically, once the tumor cells enter into an established state of senescence, ABT‐263 is used as a ‘clearing’ agent in an effort to eliminate the residual senescent tumor cells [27]. Our results indicate that ABT‐263 is effective against senescent tumor cells both *in vitro* and *in vivo*, raising the intriguing possibility of incorporating senolytics into conventional therapeutics with the goal of reducing or, ideally, eliminating residual surviving senescent tumor cells that have evaded the cytotoxicity of the primary treatments.

## 2 Materials and methods

### 2.1 Cell lines and *in vitro* drug treatments

The A549 and HEK293T cell lines were purchased from ATCC (Manassas, VA, USA), and all other cell lines were graciously provided by researchers at Virginia Commonwealth University: M. Hartman (MDA‐MB‐231) and J. Landry (Lewis Lung Carcinoma). All cell lines were maintained in DMEM (Thermo Fisher, Waltham, MA, USA) with 10% (v/v) fetal bovine serum (Gemini, West Sacramento, CA, USA), and 100 U·mL^−1^ penicillin G sodium/100 µg·mL^−1^ streptomycin sulfate (Thermo Fisher). Etoposide (Sigma‐Aldrich, St. Louis, MO, USA), doxorubicin (Tocris, Minneapolis, MN, USA), ABT‐263 (AbbVie), ABT‐199 (APExBio, Houston, TX, USA), and A‐1155463 (APExBio) were all dissolved in DMSO and administered in the dark at the desired concentrations. Radiation was performed using a ^137^Cs irradiator. To establish knockdown cell lines, viral particles were produced by triple transfection of the appropriate shRNA plasmids, psPAX2, and pMD2.G (Addgene, Watertown, MA, USA) with EndoFectin‐Lenti (GeneCopoeia, Rockville, MD, USA) into HEK293T cells. Target cells were transduced with the viral supernatant and then, where appropriate, selected for by 1 µg·mL^−1^ puromycin.

### 2.2 Antibodies

The following primary antibodies were used in these studies: lamin B1 (Cell Signaling, Danvers, MA, USA), cleaved caspase 3 (Cell Signaling), cleaved PARP (Thermo Fisher), BCL‐X_L_ (Cell Signaling), BCL‐2 (Abcam, Cambridge, UK), BAX (Cell Signaling), BAK (Cell Signaling), H3K9Me3 (Abcam), and GAPDH (Cell Signaling). The secondary antibodies used were as follows: anti‐rabbit IgG conformation‐specific (Cell Signaling), anti‐mouse‐HRP conjugated (Cell Signaling), anti‐rabbit‐HRP conjugated (Cell Signaling), anti‐rabbit‐AlexaFluor 488 (Thermo Fisher), and anti‐rabbit‐AlexaFluor 568 (Thermo Fisher).

### 2.3 Cell viability

Viable cell counts were obtained by hemocytometer at various time points during and/or after treatment. Media was replenished every 48 h.

### 2.4 SA‐β‐galactosidase staining and C_12_FDG quantification

Histochemical staining of SA‐β‐galactosidase by X‐Gal, quantification of SA‐β‐galactosidase positive cells by C_12_FDG flow cytometry, and C_12_FDG FACS were performed as described previously [10, 28, 29]. For X‐Gal staining of tissue, slices were fixed and stained by the same protocol that was used for analyzing cell culture. All images were taken on an Olympus (Tokyo, Japan) inverted microscope at 20×.

### 2.5 Cell cycle analysis and Annexin‐V/PI apoptosis staining

Cell cycle assessment (based on Propidium Iodide) and apoptosis quantification (based on Annexin‐V/PI) by flow cytometry were conducted as described previously [10, 11].

### 2.6 Immunofluorescence and immunohistochemistry

H3K9Me3 immunofluorescence was performed as previously described [10]. For cleaved caspase‐3 immunohistochemistry, tissues were fixed in cold acetone for 10 min and then blocked for 1 h at room temperature with 10% BSA. Slides were then stained overnight at 4 °C with the primary antibody at 1 : 300 and then for 2 h at room temperature with the secondary antibody (1 : 1000). Slides were mounted with Fluoroshield DAPI‐containing mounting solution (Abcam). Images were taken on an Olympus inverted microscope at 100× for H3K9Me3 and at 20× for cleaved caspase 3.

### 2.7 Western blotting

Western blots were generated as previously described [30].

### 2.8 Co‐immunoprecipitation

Equal amounts of protein lysates were incubated with the primary antibody at 4 °C overnight. Protein A/G beads (Thermo Fisher) were then added for 1 h at 4 °C to precipitate the protein–antibody complexes. Beads were centrifuged, washed, and suspended in 50/50 CHAPS buffer and 2× SDS‐loading buffer. Samples were boiled, and then, equal volumes were loaded onto an SDS/PAGE gel. Western blotting was performed as previously described [30]. Because BAX, BAK, and BCL‐X_L_ are near the IgG light chain, the IP membranes were incubated with anti‐rabbit conformation‐specific antibody (Cell Signaling) between primary and secondary blotting. Nonprecipitated samples (inputs) were processed in a similar manner, but without the conformation‐specific antibody.

### 2.9 qRT‐PCR

RNA purification and real‐time PCR were performed as described previously [10]. QuantiTect primers were purchased from Qiagen (Germantown, MD, USA): CXCL8: QT0000322; IL‐6: QT00083720; IL‐1B: QT00021385; MMP3: QT00060025; GAPDH: QT00079247. Relative mRNA expression was determined using the ∆∆*C*_t_ method.

### 2.10 High‐speed live cell interferometry

The high‐speed live cell interferometry (HSLCI) system has previously been described in detail [31]; briefly, a wide field phase detection camera is coupled to light emitting diode, motorized stages holding a 24‐well plate, and a piezo actuated autofocusing system. The detected phase change in the light is then used to calculate the mass of single cells and cell clusters. For these experiments, MDA‐MB‐231 or A549 cells were plated in a 24‐well optical glass‐bottomed plate (Cellvis, Mountain View, CA, USA) at 1–5 × 10^4^ cells per well and allowed to adhere. Cells were then exposed to 8.7 µm of etoposide for 3 days (A549) or 0.75 µm doxorubicin (MDA‐MB‐231) for 2 h. ABT‐263 was given the same day as removal of etoposide or 4 days after the removal doxorubicin. After ABT‐263 dosing, the plate was placed inside the HSLCI for imaging for 14 h at 20×, 37 °C, and 5% CO_2_. When using sorted cells, high‐C_12_FDG cells were plated and allowed to adhere overnight, followed by monitoring in the HSLCI for 14 h at 20×, 37 °C, and 5% CO_2_. Cell mass is tracked from image to image either individually or in a cluster as described previously [10].

### 2.11 Tumor‐bearing animal studies

All animal studies were conducted in accordance with Virginia Commonwealth University IACUC guidelines. To establish tumors, 2.5 million cells suspended in sterile 50/50 PBS‐Geltrex basement membrane matrix (Thermo Fisher) were injected either subcutaneously into both rear flanks (A549 cells, male NSG) or orthotopically by surgical implantation into both a left and right mammary fat pad (MDA‐MB‐231 cells, female NSG). Once tumors approached 100 mm^3^, A549‐tumor‐bearing mice were treated with 15 mg·kg^−1^ etoposide (Massey Cancer Center Pharmacy, Richmond, VA, USA, diluted in 80% PBS, 10% ethanol, 5% DMSO, 5% emulphor v/v) by intraperitoneal injections every other day for a total of five injections. MDA‐MB‐231 tumor‐bearing mice were treated with 2.5 mg·kg^−1^ doxorubicin (Massey Cancer Center Pharmacy) by intraperitoneal injections once weekly for a total of two injections. Cohorts that received ABT‐263 (AbbVie) were administered 50 mg·kg^−1^ (dissolved in 60% phosal, 30% PEG, 10% ethanol v/v) by oral gavage every other day for a total of 7 days, beginning 24 h after last chemotherapy treatment. Tumor volumes were taken by manual caliper measurements. For X‐Gal and immunofluorescence staining, tumors were frozen into OCT molds and cut into 5‐µm sections by the Tissue and Data Acquisition and Analysis Core at VCU.

### 2.12 Statistical analysis

Unless otherwise indicated, all quantitative data are shown as mean ± SEM from at least three independent experiments, all of which were conducted in triplicates or duplicates. graphpad prism 6.0software (San Diego, CA, USA) was used for statistical analysis. All data were analyzed using either a one‐ or two‐way ANOVA, as appropriate, with Tukey or Sidak *post hoc* as appropriate, with the exception of the following: C_12_FDG data with only two groups were analyzed with unpaired, Student's *t*‐tests; HSLCI obtained median masses for each group was compared pairwise to the control group using Mood's median test where each trial was considered as one replicate.

## 3 Results

### 3.1 Induction of senescence in tumor cell models in response to etoposide, doxorubicin, and radiation

**Fig. 1:**
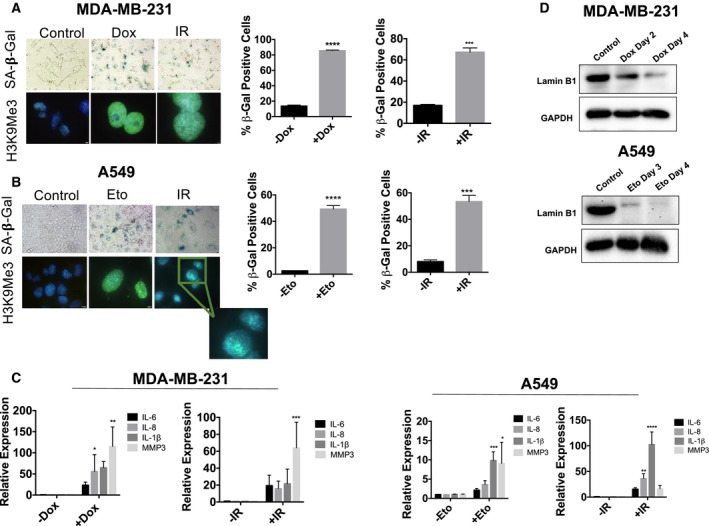
Topoisomerase poisons (Dox and Eto) and radiation induce senescence in breast and lung cancer cell lines. For all the following experiments MDA‐MB‐231 and A549 cells exposed to either Dox (750 nm for 2 h), Eto (8.7 µm for 72 h), or IR (8 or 10 Gy). (A, B) Dox‐ or IR‐treated MDA‐MB‐231 cells and Eto‐ or IR‐treated A549 cells were evaluated for increased expression of SA‐β‐gal using X‐gal (bright‐field images, objective 20×) or C_12_FDG (bar graphs) and increased SAHF formation by H3K9ME3 immunofluorescence (fluorescent images, objective 100× oil immersion). The IR image for H3K9Me3 in A549 cells has been expanded for ease of visualization. Staining was performed at Day 4 for MDA‐MB‐231 cells with both Dox and IR, and Day 3 for A549 cells with both Eto and IR. Blue fluorescence indicates nuclear staining with DAPI, and green fluorescence reflects H3K9Me3 immunostaining. ***P ≤ 0.001 and ****P ≤ 0.0001 indicate statistical significance of each condition compared to controls (Eto, Dox, and IR) as determined using unpaired, Student's *t*‐test. (C) qRT‐PCR for the SASP mRNAs *IL‐6, IL‐8, IL‐1β*, and *MMP3* was performed following drug or radiation exposure. RNA extraction was performed at Day 4 following exposure for MDA‐MB‐231 cells and Day 3 following exposure for A549 cells. *P ≤ 0.05, **P ≤ 0.01, and ***P ≤ 0.001 indicate statistical significance of each condition compared to control (Eto, Dox, and IR) as determined using two‐way ANOVA with Sidak's *post hoc* test. (D) Western blotting for Lamin B1 in MDA‐MB‐231 cells (left) and A549 cells (right) following treatment with Dox or Eto, respectively. All images are representative fields or blots from three independent experiments (*n* = 3), and all quantitative graphs are mean ± SEM from three independent experiments (*n* = 3).

Senescence is an established response to genotoxic therapies in tumor cells [32]. Topoisomerase poisons such as doxorubicin and etoposide as well as radiation represent treatment modalities that are known to act via DNA damage (reviewed in Ref. [33]). As such, we induced senescence in non‐small‐cell lung carcinoma cells (A549) and triple‐negative breast cancer cells (MDA‐MB‐231) by treatment with either etoposide (Eto) or doxorubicin (Dox), respectively, or ionizing radiation (IR). Based on pharmacokinetic data, cells were exposed to Eto at a concentration of 8.7 µm for 72 h or to Dox at 750 nm for 2 h [34, 35]. Ionizing radiation was administered at supraclinical doses of 10 Gy for A549 cells and 8 Gy for MDA‐MB‐231 cells for proof of concept relating to senescence induction. Figure 1A,B illustrates promotion of senescence by doxorubicin, etoposide, and radiation based on increased SA‐β‐gal activity, morphological alterations (enlargement, flattening, and granulation), and heterochromatic H3K9Me3 foci formation. Drug and IR exposure also increased transcriptional expression of multiple SASP components, including IL‐1β, IL‐6, IL‐8, and MMP3 [2] (Fig. 1C). Degradation of Lamin B1, another indication of senescence, was also detected in cells treated by doxorubicin or etoposide [36] (Fig. 1D). Collectively, these results indicate that exposure of breast and lung tumor cells to either topoisomerase poisons or radiation results in a senescent growth arrest.

### 3.2 ABT‐263 selectively eliminates senescent tumor cells *in vitro*

**Fig. 2:**
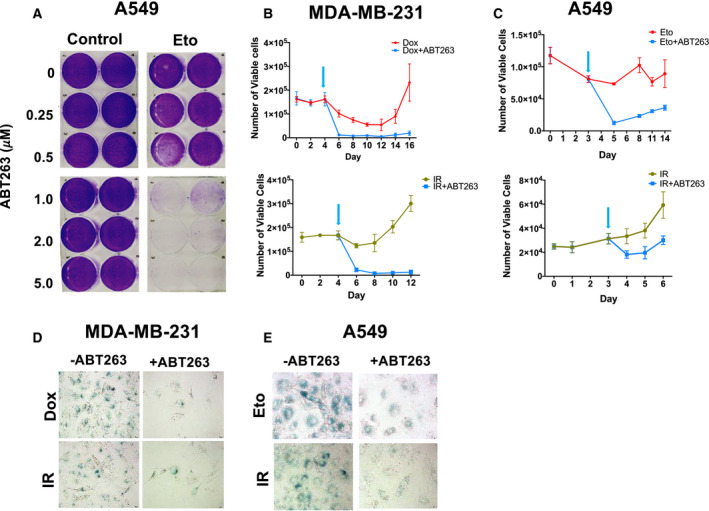
ABT‐263 is capable of clearing senescent cells after drug or radiation treatment. (A) Crystal violet assay showing a dose response for ABT‐263's effect on proliferating or Eto‐induced senescent A549 cells. A549 cells were treated with vehicle or 8.7 µm Eto for 72 h, and then exposed to the indicated concentrations of ABT‐263 for 48 h. (B, C) MDA‐MB‐231 (B) and A549 (C) growth curves for cells treated with Dox/Eto (top panel) or IR (bottom panel), followed by either vehicle or 2 µm ABT‐263 for 48 h. Arrows indicate ABT‐263 treatment start. (D, E) X‐gal staining for SA‐β‐gal positive cells after ABT‐263 exposure in drug‐treated (top panels) or IR‐exposed (bottom panels) MDA‐MB‐231 (D) and A549 cells (E). All bright field images were captured at the same magnification (objective 20×) and are representative fields from three independent experiments (*n* = 3), and all quantitative graphs are mean ± SEM from at three independent experiments (*n* = 3).

To confirm the senolytic potential of ABT‐263, the senescent tumor cells generated by the previous treatments were exposed to ABT‐263 and the surviving populations were stained with crystal violet. ABT‐263 treatment resulted in robust elimination of senescent, but not proliferating, A549 cells (Fig. 2A). Likewise, a single 48‐h exposure to 2 µm ABT‐263 significantly reduced viable cell number in senescent Eto‐, Dox‐, or IR‐treated cells (Fig. 2B,C), but not in untreated controls (Fig. S1A,B). ABT‐263 markedly reduced the number of SA‐β‐gal‐positive cells, resulting in a population with minimal or no X‐Gal staining (Fig. 2D,E); furthermore, the capacity of ABT‐263 to drive the Eto‐treated A549 cells toward cell death diminished over time as the cells recovered from senescence (Fig. S1C). This is a critical observation that may provide guidance as to the optimal intervals during which to administer senolytic agents in the clinic.

**Fig. 3:**
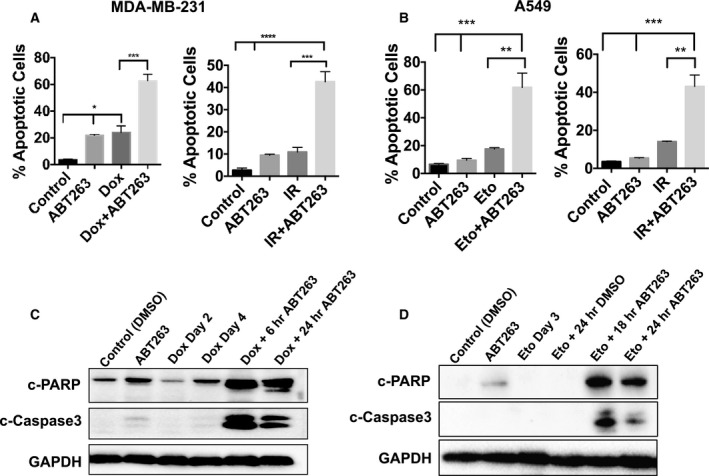
ABT‐263 induces apoptotic cell death in senescent cells. (A, B) Annexin‐V/PI quantification of apoptosis induced by 2 µm ABT‐263 with overnight exposure in MDA‐MB‐231 (A) or A549 (B) cells after treatment with Dox/Eto (left panels) or IR (right panels). *P ≤ 0.05, **P ≤ 0.01, ***P ≤ 0.001, and ****P ≤ 0.0001 indicate statistical significance comparing the bracketed conditions as determined using one‐way ANOVA with Tukey's *post hoc* test. (C, D) Western blotting for cleaved PARP and cleaved caspase‐3 in MDA‐MB‐231 (C) and A549 (D) cells for the indicated treatments and time points. All images are representative fields or blots from three independent experiments (*n* = 3), and all quantitative graphs are mean ± SEM from three independent experiments (*n* = 3).

As expected from previous studies involving senolytics and tumor cells [15, 27, 37], ABT‐263 exerts its effects against senescent tumor cells by inducing apoptotic cell death, as demonstrated by the increase in Annexin‐V/PI staining and the apoptosis markers cleaved PARP and cleaved caspase‐3 (Fig. 3A–D). Again, these results were only evident in senescent cells (Fig. 3A–D, comparing ABT‐263 alone to treatment + ABT‐263 conditions). Similarly, ABT‐263 promoted apoptotic cell death (Fig. S2C–E) in murine Lewis lung carcinoma (LLC) cells induced into senescence by etoposide (Fig. S2A), but not in nonsenescent control cells (Fig. S2B). Figure S2F shows the reduction in senescent tumor cells after treatment with ABT‐263. Taken together, these data confirm that ABT‐263 acts as a selective senolytic *in vitro*, effectively reducing the number of tumor cells after induction into senescence by chemotherapy or radiation.

### 3.3 Cells that escape ABT‐263‐induced cell death are likely not senescent

We previously reported that HSLCI can differentiate between senescent and nonsenescent cells based on cellular mass and changes in mass over time [10]. To investigate which cells specifically responded to ABT‐263 within the Eto‐ or Dox‐treated populations, HSLCI was employed to monitor biomass changes of individual cells during ABT‐263 treatment. The addition of ABT‐263 depressed median growth to 0% or below, indicating loss of mass and therefore cell death, only in cells pretreated with Eto or Dox (Fig. S3A,B), as shown previously. However, a small population within the Eto‐ and Dox‐treated populations maintained a positive growth rate, indicating that some cells had escaped the effects of ABT‐263 (Fig. S3A,B). Determination of the single cell mass at the initiation of ABT exposure (time 0) confirmed our previous finding that SA‐β‐gal‐positive cells are significantly larger than either control or SA‐β‐gal‐negative cells [10] (Table 1). A549 median control cell mass ranged from 963 ± 90 pg to 1047 ± 56 pg, while the median SA‐β‐gal‐positive cell mass ranged from 1356 ± 186 pg to 1818 ± 114 pg. MDA‐MB‐231 median control cell mass ranged from 730 ± 36 pg to 900 ± 13 pg, while the median SA‐β‐gal‐positive cell mass ranged from 1124 ± 102 pg to 1414 ± 139 pg. For MDA‐MB‐231 cells treated with Dox + ABT‐263, the top 10% of growing cells (cells that do not die with ABT‐263) were consistently smaller than SA‐β‐gal‐positive cells, while the bottom 10% of cells by growth rate (dying cells) were the same size (Fig. S3D and Table 1). These cell size measurements suggest that the cells that are unaffected by ABT‐263 are likely not senescent. Similar trends were observed in Eto + ABT‐263‐treated A549 cells (Fig. S3C and Table 1). Overall, these size analyses emphasize ABT‐263 specificity, as ABT‐263 affects characteristically large senescent cells while sparing smaller cells which have not undergone senescence as a response to the topoisomerase inhibitors.

**Table 1:** Determination of single cell mass values of A549 and MDA‐MB‐231 in the senescent and nonsenescent states. The median single cell mass for each condition was determined by identifying the number of nuclei, typically ranging from one to five, in each cell cluster for which biomass was successfully tracked. The median was then taken of the population of mean single cell masses of all tracked clusters. The table shows median cell mass values and their corresponding 95% confidence intervals for each trial and treatment condition.

A. MDA‐MB‐231						
Trial #	Vehicle	ABT263	Dox	C_12_FDG High	Top 10% (Growing)	Bottom 10% (Dying)
1	900 ± 13	916 ± 10	1272 ± 72	1124 ± 72	572 ± 102	1280 ± 142
2	817 ± 37	784 ± 42	1495 ± 273	1414 ± 39	504 ± 125	1158 ± 137
3	730 ± 36	669 ± 16	1162 ± 86	ND	645 ± 96	1695 ± 87

B. A549						
Trial #	Vehicle	ABT263	Eto	C_12_FDG High	Top 10% (Growing)	Bottom 10% (Dying)
1	963 ± 91	ND	1400 ± 100	1356 ± 186	334 ± 122	1562 ± 195
2	1047 ± 56	1103 ± 50	1423 ± 146	1818 ± 114	734 ± 186	2553 ± 160
3	1026 ± 139	1087 ± 74	1960 ± 90	ND	1821 ± 110	1921 ± 78

### 3.4 ABT‐263 reduces tumor burden of chemotherapy‐treated tumor‐bearing animals and interferes with the tumor‐initiating potential of senescent tumor cells

**Fig. 4:**
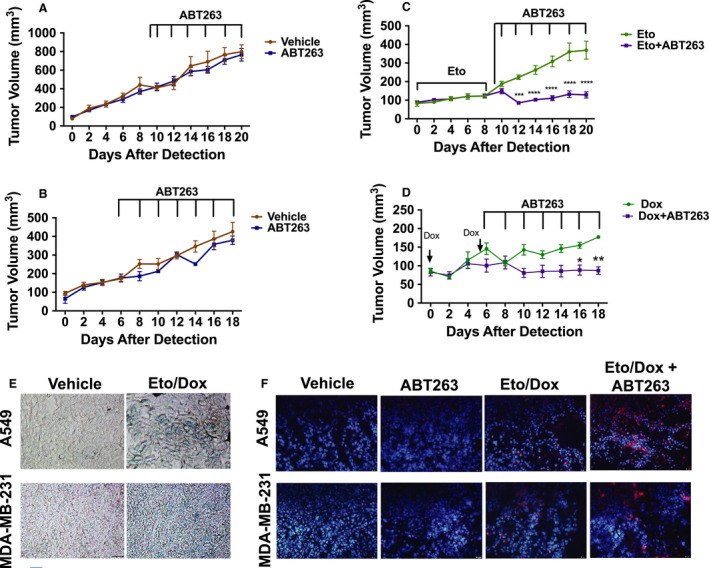
Sequential administration of ABT‐263 following chemotherapy confers decreased tumor burden *in vivo*. (A, B) Tumor volume over time in A549 (A) and MDA‐MB‐231 (B) tumor‐bearing mice that received either vehicle or ABT‐263 (50 mg·kg^−1^) only. (C, D) Tumor volume over time in A549 (C) and MDA‐MB‐231 (D) tumor‐bearing mice that received either chemotherapy (15 mg·kg^−1^ Eto or 2.5 mg·kg^−1^ Dox) only or chemotherapy followed by ABT‐263 (50 mg·kg^−1^). ***P ≤ 0.001 and ****P ≤ 0.0001 indicate statistical significance of tumor volumes of mice treated with Eto alone vs. tumor volumes of mice treated with Eto + ABT263 (C) as determined using two‐way ANOVA with Sidak's *post hoc* test, while *P ≤ 0.05 and **P ≤ 0.01 indicate statistical significance of tumor volumes of mice treated with Dox alone vs. tumor volumes of mice treated with Dox + ABT263 (D) as determined using two‐way ANOVA with Sidak *post hoc* test. (E) X‐Gal staining of tumor slices from mice in the indicated groups. All bright‐field images were captured at the same magnification (objective 20×). (F) Immunofluorescence for cleaved caspase‐3 and DAPI in tumor slices from mice in the indicated groups. Blue fluorescence indicates nuclear staining with DAPI, and red fluorescence reflects caspase 3 immunostaining. All fluorescent images were captured at the same magnification (objective 20×). A549 tumor volumes, *n* = 4 for vehicle, *n* = 8 for ABT‐263, *n* = 9 for Eto, and *n* = 14 for Eto + ABT‐263. For MDA‐MB‐231 tumor volumes, *n* = 4 for vehicle, *n* = 4 for ABT‐263, *n* = 5 for Dox, and *n* = 6 for Dox + ABT‐263 (n denotes number of mice harboring bilateral tumors). Graphs are represented as mean ± SEM. All tumor images are representative fields from four tumor slices (*n* = 4) taken from two mice per group (*n* = 2).

We next extended our findings of ABT‐263 administered subsequent to senescence‐inducing chemotherapy to assess the effects on tumor burden *in vivo* in studies where A549 or MDA‐MB‐231 cells were implanted in mice (Fig. 4). Once implanted, and tumors reached ~ 100 mm^3^, mice challenged with A549 cells were exposed to Eto (15 mg·kg^−1^, 5 injections), while mice challenged with MDA‐MB‐231 cells were exposed to Dox (2.5 mg·kg^−1^, 2 injections). Both treatments resulted in tumor stasis (Fig. 4C,D) and the induction of intratumoral senescence as marked by increased SA‐β‐gal activity in comparison with vehicle‐treated mice (Fig. 4E). Of note, static tumor volume was followed by proliferative recovery, typical of the tumor growth delay routinely observed in studies of chemotherapy or radiation treatment of tumor‐bearing animals.

After senescence establishment and cessation of chemotherapy, mice were treated with ABT‐263 (50 mg·kg^−1^) by oral gavage every other day for a total of 7 administrations. While ABT‐263 exerted no effect on tumor growth as a monotherapy (Fig. 4A,B), the sequential treatment with ABT‐263 resulted in a clear therapeutic benefit marked by a significant decrease in tumor volume and prolonged tumor maintenance (Fig. 4C,D). Notably, in both A549 and MDA‐MB‐231 tumor‐bearing mice, sequential administration of ABT‐263 interfered with the recovery seen in mice that received chemotherapy alone. This is a critical observation that suggests that this sequential therapeutic strategy could directly influence disease recurrence. Finally, *ex vivo* analysis of apoptosis, as judged by cleaved caspase‐3 immunofluorescence, indicated that only the sequential treatment, but neither chemotherapy nor ABT‐263 alone, promoted apoptosis (Fig. 4F). This suggests that the reduction in tumor burden was likely a consequence of driving senescent tumor cells into apoptotic cell death. Collectively, these results indicate that ABT‐263 interferes with proliferative recovery from the senescent state by directly inducing cell killing of senescent cells and could potentially abet the response to conventional therapeutics.

### 3.5 ABT‐263 exerts its senolytic activity in senescent tumor cells by inhibiting BCL‐X_L_'s interaction with BAX

Members of the BCL‐2 family regulate the survival of different types of malignant cells and have also been shown to mediate the resistance of senescent cells to cell death [38, 39]. ABT‐263 is a BH3 mimetic that inhibits anti‐apoptotic BCL‐2 family proteins by impeding their ability to bind pro‐apoptotic proteins, such as BAK and BAX [40]. BCL‐2 and BCL‐X_L_ are the primary targets of ABT‐263 in cancer cells [41]. We therefore sought to determine which of these proteins acts as ABT‐263's functional target during chemotherapy‐induced senescence.

**Fig. 5:**
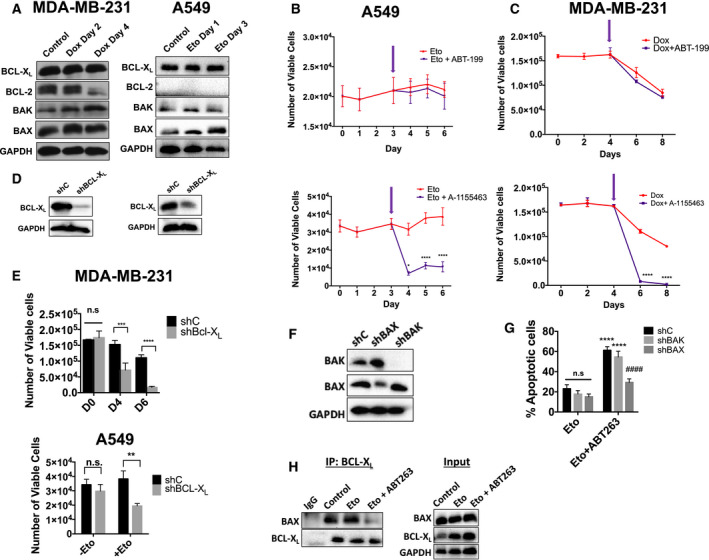
ABT‐263 induces apoptosis by disrupting BCL‐X_L_'s interaction with BAX. (A) For all the following experiments, MDA‐MB‐231 and A549 cells exposed to either Dox (750 nm for 2 h) or Eto (8.7 µm for 72 h), respectively. Western blot analysis for BCL‐2 family members in MDA‐MB‐231 and A549 cells during treatment with Dox or Eto, respectively. (B, C) Viable cell number for A549 cells treated with Eto (B) or MDA‐MB‐231 cells treated with Dox (C) followed by 2 µm ABT‐199 (top panels) or 2 µm A‐1155463 (bottom panels). Arrows indicate time at which ABT‐199 or A‐1155463 treatment started.). *P ≤ 0.05 and ****P ≤ 0.0001 indicate statistical significance comparing Eto/Dox vs. Eto/Dox + ABT199 or Eto/Dox vs. Eto/Dox + A‐1155463 as determined using two‐way ANOVA with Sidak's *post hoc* test. (D) Western blots for shBCL‐X_L_ knockdown cells. (E) Viable cell number for shC and shBCL‐X_L_ cells following treatment with Dox or Eto.***P ≤ 0.001 and ****P ≤ 0.0001, n.s. (not significant), indicate statistical significance comparing viable cell number of shC vs. shBCL‐X_L_ variants as determined using two‐way ANOVA with Sidak's *post hoc* test (for MDA‐MB‐231 cells) or an unpaired, Student's *t*‐test (for A549 cells). (F) Western blot for BAK and BAX in A549 shC, shBAK, and shBAX cells. (G) Annexin‐V/PI flow cytometry for A549 shC, shBAX, and shBAK cells treated with Eto or Eto + ABT‐263. ****P ≤ 0.0001 indicates statistical significance compared to Eto‐only treated cells, ^####^ *P* ≤ 0.001 indicates statistical significance compared to shC variants within treatment group and n.s. indicates no statistical significance all as determined by a two‐way ANOVA with Sidak's *post hoc* test (H) Co‐immunoprecipitation assays against BCL‐X_L_ and the corresponding inputs for A549 cells for the indicated conditions. All images are representative fields or blots from three independent experiments (*n* = 3), and all quantitative graphs are mean ± SEM from three independent experiments (*n* = 3).

Western blot analysis of these anti‐apoptotic proteins demonstrated that BCL‐X_L_ expression was consistently high in both MDA‐MB‐231 and A549 cells. In contrast, BCL‐2 expression was gradually decreased in MDA‐MB‐231 cells or undetectable in A549 cells (Fig. 5A). These results suggest that BCL‐X_L_ might play a direct role in mediating the survival of senescent cells. To further test this hypothesis, we compared the impact on apoptosis in senescent cells of the BCL‐X_L_‐specific inhibitor, A‐1155463 [40], and the BCL‐2‐specific inhibitor, ABT‐199 [27]. As with ABT‐263, neither inhibitor had an effect on proliferating, nonsenescent cells (Fig. S4A,B). In both Eto‐ and Dox‐induced senescent cells, however, A‐1155463 significantly reduced viable cell number while ABT‐199 showed minimal effect (Fig. 5B,C). We further substantiated the effects of BCL‐X_L_ inhibition with shRNA knockdown. MDA‐MB‐231 cells were stably transduced with shC (scrambled sequence) or shBCL‐X_L_ (Fig. 5D), and then exposed to Dox. shC cells responded similarly to nontransduced cells following treatment with Dox, while shBCL‐X_L_ cells underwent a dramatic decrease in viability (Fig. 5E), following senescence induction at day 4 (Fig. S4C). A549 cells could not survive a stable transduction of shBCL‐X_L_ (data not shown), and as such, A549 cells were first induced into senescence by Eto and then transduced with shC or shBCL‐X_L_ (Fig. 5D). As with MDA‐MB‐231 cells, shBCL‐X_L_ caused a decline in cell viability compared to shC in senescent conditions only (Fig. 5E). Altogether, these data suggest that cells induced into senescence by either Dox or Eto become dependent upon BCL‐X_L_, but not BCL‐2 for survival, and therefore, ABT‐263 likely initiates senolysis via its inhibition of BCL‐X_L_.

Inhibition of BCL‐X_L _by ABT‐263 should result in BAX and/or BAK activation. To confirm the involvement of these executioner proteins following ABT‐263 treatment, we established stable shC, shBAX, and shBAK A549 cells (Fig. 5F). Importantly, all of these cell lines undergo similar amounts of senescence following exposure to Eto (Fig. S4D). When these stable knockdown cells were exposed to Eto followed by ABT‐263, shC‐ and shBAK‐expressing cells underwent apoptotic cell death, as indicated by increased Annexin‐V‐positive cells and decreased number of viable cells (Fig. 5G and Fig. S4E). However, shBAX‐expressing cells failed to undergo apoptosis following exposure to ABT‐263 (Fig. 5G and Fig. S4E), indicating that BAX is essential to ABT‐263's mechanism.

BCL‐X_L_ has previously been shown to bind BAX directly [42], and this interaction should be disrupted by ABT‐263. Co‐immunoprecipitation assays (Fig. 5H) confirmed that in the nonsenescent and senescent states, BAX co‐precipitates with BCL‐X_L_. Addition of ABT‐263 decreased co‐precipitation of BAX, suggesting disruption of its binding to BCL‐X_L_. Taken together, these data demonstrate that ABT‐263‐induced apoptosis in senescent cells specifically occurs via the disruption of the BAX/BCL‐X_L_ complex.

## 4 Discussion

Although TIS has been studied for decades, it remains uncertain how the senescent response contributes to (or interferes with) disease control [43]. Senescence has been proposed as a favorable outcome of cancer treatment since senescent tumor cells are in a growth‐abrogated phase, and, accordingly, development of novel therapeutics that can induce senescence in tumor cells has been encouraged [44]. However, this rationale has generally neglected to consider the extensive heterogeneity of senescent tumor cells at both the genomic and transcriptomic levels [45, 46], and how they senesce differently in response to therapy [47], thus overlooking the possibility that the senescent growth arrest might not be obligatorily permanent. In addition, senescent cells are by definition resistant to apoptosis, persistent, and metabolically active [3]. Moreover, recent studies have reported that cells derived from a senescent tumor population are often more aggressive than the original population [48, 49], which would be consistent with the difficulties encountered when treating recurrent cancer. This accumulating evidence provided the incentive for our current efforts to explore preclinical approaches to target and eliminate senescent tumor cells [6].

In this context, several senolytic tools have been considered to eliminate senescent tumor cells. For example, an experimental cytotoxic drug delivery system based on the upregulation of SA‐β‐gal cleared senescent tumor cells induced by the CDK4/6 inhibitor, palbociclib, and resulted in dramatic tumor xenograft regression [50]. In addition, the serotonin‐selective reuptake inhibitor (SSRI) sertraline has been shown to selectively kill senescent liver cancer cells as part of a drug screening for novel senolytic compounds [51]. Moreover, cardiac glycosides, such as digoxin or ouabain, have been shown to kill senescent tumor cells, albeit at supraclinical concentrations [52, 53]. Lastly, panobinostat, a histone deacetylase inhibitor, evidently eliminated lung and head and neck tumor cells induced into senescence by cisplatin or paclitaxel *in vitro* [54].

To date, the most comprehensively studied senolytic agent so far is the established BCL‐2/BCL‐X_L_ inhibitor, ABT‐263 (navitoclax). We here present evidence for a sequential treatment approach that exploits ABT‐263's senolytic potential against senescent tumor cells. ABT‐263 has previously shown superiority in eliminating senescent tumor cells (either induced into senescence by topoisomerase II or aurora kinase inhibition) over other senolytic modalities such as the combination of dasatinib + quercetin, which is efficacious in killing nontumor senescent cells [27, 55]. Our data indicate that ABT‐263 is highly efficient in inducing selective cell death in senescent tumor cells, similar to its action on other non‐tumor‐senescent cell types [25, 38, 56, 57, 58, 59]. Similar to our results, a report by Fleury *et al*. [60] has demonstrated that exposure of p53‐mutated ovarian and triple‐negative breast tumor cells to the PARP inhibitor, olaparib, results in the induction of *reversible* senescence. Interestingly, olaparib‐induced senescent tumor cells were selectively eliminated by ABT‐263 exposure when applied prior to their recovery from senescence. The senolytic ability of ABT‐263 was also dependent on BCL‐X_L_‐mediated resistance to apoptosis [60]. Finally, our data support the premise of the ‘two‐hit’ approach as an effective anticancer strategy [61, 62] that may prevent disease recurrence by interfering with the ability of growth‐arrested tumors to recover. The ‘two‐hit’ approach can potentially serve parallel adventitious effects, as the use of senolytics, including ABT‐263, is expected to also eliminate other senescent, but nonmalignant, cells. Evidence has shown that exposing IMR90 human lung fibroblasts to 20 µm etoposide induces senescence in 70% of the cell population and that these senescent fibroblasts are amenable to clearance by 17‐DMAG, an HSP90 inhibitor senolytic [63]. Additionally, systemic treatment of mice with doxorubicin was shown to increase the burden of non‐tumor‐senescent cells, which were also subject for elimination by ABT‐263 [15]. Accordingly, nontumor cells are also expected to senesce in response to genotoxic stress, albeit less readily than tumor cells, and thus, the elimination of non‐tumor‐senescent cells is not only expected to interfere with co‐existing aging processes [64], but also to perturb tumorigenic properties associated with the accumulation of senescent cells. This expectation is in part based on evidence that the SASP drives deleterious paracrine influence that promotes several adverse effects associated with conventional chemotherapy, which may include disease relapse [15]. Also, a pro‐tumor SASP response could lead to a suppressed immune response and the maintenance of dormant tumor cells [65, 66, 67].

As a senolytic, ABT‐263 efficiently induces apoptosis only in therapy‐induced senescent cells, but not in proliferating cells (Fig. 2 and Fig. S1). We found that the inhibition of BCL‐X_L_/BAX interaction by ABT‐263 is critical for this apoptotic mechanism (Fig. 5). A gradual increase of BCL‐X_L_ expression during the senescent state and lack of dependency of most solid tumors on BCL‐2 for survival would explain the BCL‐X_L_ dependency [40]. This dependency has also been observed in cisplatin‐treated and radiation‐induced senescent head and neck squamous cell carcinoma cell lines (H. Harada unpublished data), suggesting the possibility that different solid tumors that undergo DNA damage‐induced senescence could be susceptible to ABT‐263. The role of BCL‐X_L_ in apoptosis resistance of senescent tumor cells was further supported by studies by Gayle *et al*. [68] showing that senescent, triple‐negative breast tumor cells induced by bromodomain and extra‐terminal proteins (BET) inhibitors express higher levels of BCL‐X_L_. Furthermore, while BCL‐X_L_ overexpression confers resistance of senescent tumor cells to cell death, pharmacological and genetic inhibition of BCL‐X_L_ force them into mitotic catastrophe [68]. In contrast, it is still unclear why BAX, but not BAK, plays a role in the observed apoptosis, although both proteins can equally interact with BCL‐X_L_. This question needs to be clarified in further studies.

We do acknowledge some potential challenges relating to this therapeutic approach. First, ABT‐263 treatment has been associated with dose‐limiting thrombocytopenia, a direct result of BCL‐X_L_ inhibition [40]. While this toxicity can warrant dose reductions, substantial optimization of the clinical use of ABT‐263 has afforded options for managing thrombocytopenia in patients. Early studies in patients with hematological and solid tumors indicated that a subtherapeutic dose (150 mg) for 7 days caused a low level of platelet loss yet served to prime megakaryocytes for platelet production prior to therapeutically directed steady state dosing. This lead‐in dose was shown to help reduce acute platelet nadirs and grade 4 thrombocytopenia and is now used in multiple ongoing navitoclax trials [69]. Additionally, dose optimization of navitoclax has demonstrated the ability to combine this agent with relevant therapies in a manner that enables the safe administration of therapy over time. Recently reported data in myelofibrosis patients, for example, demonstrated that doses in the 200–250 mg range combine with ruxolitinib to lessen disease burden and reduce spleen volumes compared to ruxolitinib alone, thus serving as a POC for BCL‐X_L_ inhibition [70]. In addition, and has also been reported, both ABT‐263 and ABT‐199 cause neutropenia in the clinic via BCL‐2 inhibition; this neutropenia can be exacerbated by concomitant treatment with chemotherapy [71]. However, the proposed strategy temporally separates exposure to ABT‐263 (or potentially other senolytics) from the time of chemotherapy, which could reduce the potential toxicity of this therapeutic combination. Finally, as shown by our data and others, there is limited potential for the less toxic BCL‐2‐selective inhibitor ABT‐199 to kill senescent cells, since senolysis is largely dependent on BCL‐X_L_ inhibition. Therefore, BCL‐X_L_ targeting agents that are more selective for tumor cells over platelets are promising strategies. For example, a recent report has identified a modified ABT‐263 small molecule (degradomer/PROTAC) that has remarkable selectivity to target BCL‐X_L_ in tumor cells, but not in platelets [72].

Second, our *in vitro* data suggest that a single exposure to ABT‐263 does not eliminate the entire senescent population and the persistent cells can also recover proliferation. This suggests that senolytic treatment should be repetitive and chronic in order to eliminate the majority of senescent tumor cells. Lastly, current senolytic therapy is unlikely to be selective for only ‘harmful’ senescent cells, and consequently, we expect some adverse reactions that are related to interference with the physiological roles of senescent cells, such as impaired wound healing [73]. Overall, while these studies provide proof‐of‐principle of the advantageous outcome of senescence clearance therapy as an approach to mitigate cancer recurrence, future studies will also be directed toward the optimization of BCL‐2/BCL‐X_L_ inhibitors such as navitoclax as well as the identification of less toxic senolytic agents. It is noteworthy that while this work was under review, a paper by Shahbandi *et al*. [74] was published that largely confirms our observations relating to the selectivity of ABT‐263 in eliminating senescent tumor cells.

## 5 Conclusion

We show here that sequential use of the senolytic, ABT‐263, reduces the surviving cell population following chemotherapy and radiation by selectively inducing apoptosis in senescent cells via a BCL‐X_L_/BAX‐dependent mechanism. This novel approach in which senolytic therapy is administered subsequent to senescence induction led to decreased tumor volume and delayed proliferative recovery *in vivo*, providing initial evidence that senolytic therapy following conventional cancer therapy may improve therapeutic outcomes and delay disease recurrence.

## Conflict of interest

JDL and AJS are employees and shareholders of AbbVie Inc. AF has been a paid consultant for AbbVie.

## Author contributions

TS, VJC, and LT‐M initiated the study designed and performed experiments, analyzed data, and wrote the manuscript. GM and JR performed HSLCI experiments, analyzed data, and wrote the manuscript. AF, JDL, AJS, and MRA revised the manuscript and contributed conceptually to experimental design. HH and DAG supervised the work, designed experiments, and wrote the manuscript.

## Supporting information


**Fig. S1.** ABT‐263 has minimal cytotoxicity on non‐senescent or recovering senescent tumor cells.

**Fig. S2.** ABT‐263 induces cell death in senescent LLC lung cancer cells after etoposide treatment.

**Fig. S3.** Cells that escape ABT‐263 effects are unlike typical senescent cells.

**Fig. S4.** ABT‐199 and A‐1155463 have no effect on control cells and ABT‐263 induces apoptosis by disrupting BCL‐XL's interaction with BAX.

## Data Availability

The raw research data are available per request through the corresponding author.
