# Supplementary material for: Clearance of therapy‐induced senescent tumor cells by the senolytic ABT‐263 via interference with BCL‐XL–BAX interaction
Source: Mol Oncol. 2020 Aug 5;14(10):2504–19. doi: 10.1002/1878-0261.12761 (PMC7530780; doi:10.1002/1878-0261.12761)
Supplement: Supplementary file 1 — Fig. S1. ABT‐263 has minimal cytotoxicity on non‐senescent or recovering senescent tumor cells. [file MOL2-14-2504-s001.pdf]

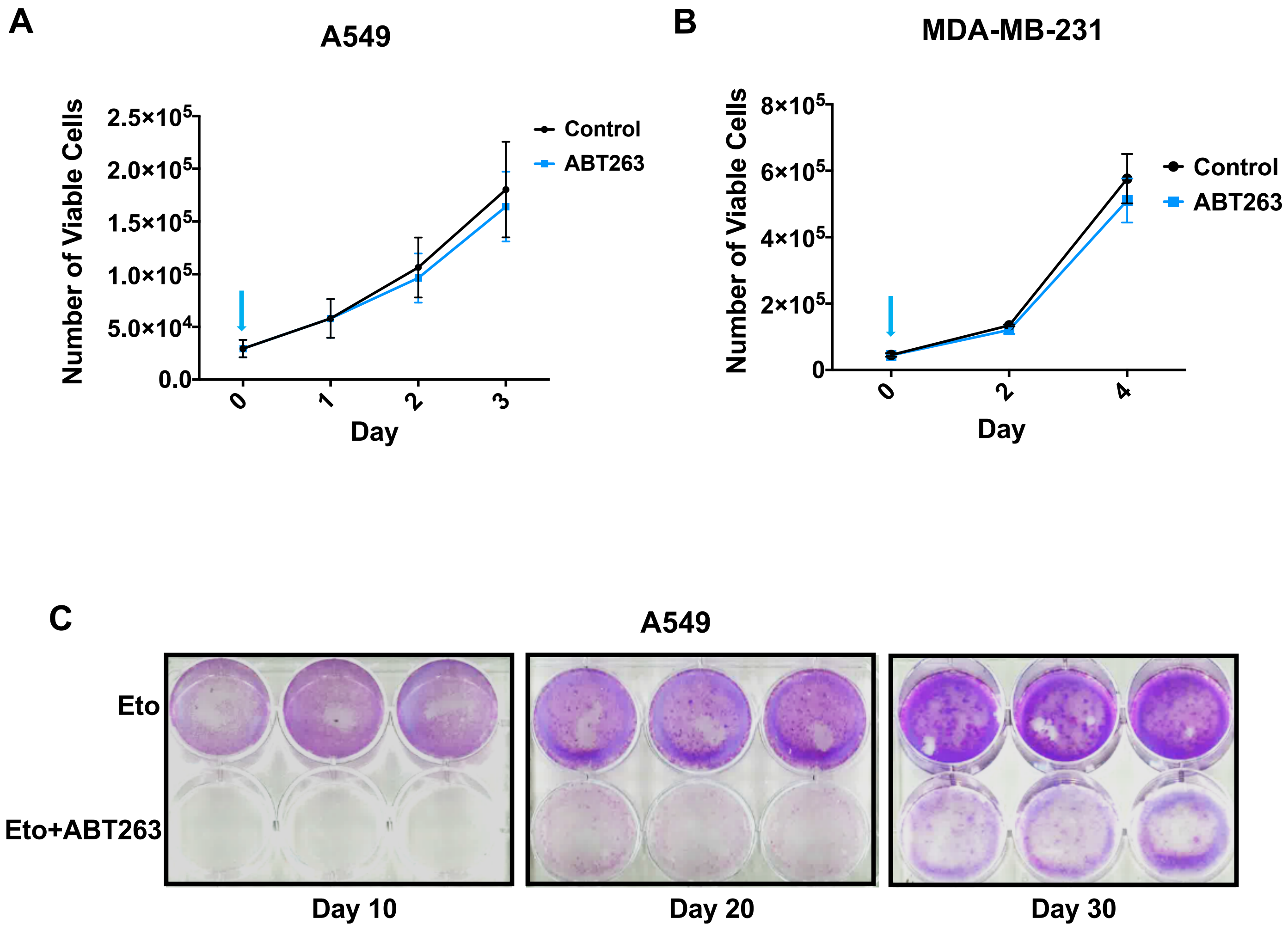
A
A549
Number of Viable Cells
2.5×10^5
2.0×10^5
1.5×10^5
1.0×10^5
5.0×10^4
0.0
0
1
2
3
Day
Control
ABT263
B
MDA-MB-231
Number of Viable Cells
8×10^5
6×10^5
4×10^5
2×10^5
0
0
2
4
Day
Control
ABT263
C
A549
Eto
Eto+ABT263
Day 10
Day 20
Day 30

Figure. S1. ABT-263 has minimal cytotoxicity on non-senescent or recovering senescent tumor cells. (A and B) Viable cell counts for A549 (A) and MDA-MB-231 (B) with or without 2 μM ABT-263. Arrows indicate start of ABT-263 treatment. No statistical significance was found as determined by two-way ANOVA with Sidak's post hoc. **(C)** Crystal violet assay showing A549 cells at the indicated time points following the end of a 7-day, continuous exposure to Eto (2 μM). Figure shows that ABT-263 continues to eliminate senescent cells up to 20 days after senescence induction and drug removal, however, its senolytic effect is diminished as tumor cells escape the senescent state as shown by the reduced cell killing at day 30. All figures are mean ± SEM three independent experiments (n=3). (C) is a representative image from three independent experiments (n=3).
