# Supplementary material for: Clearance of therapy‐induced senescent tumor cells by the senolytic ABT‐263 via interference with BCL‐XL–BAX interaction
Source: Mol Oncol. 2020 Aug 5;14(10):2504–19. doi: 10.1002/1878-0261.12761 (PMC7530780; doi:10.1002/1878-0261.12761)
Supplement: Supplementary file 2 — Fig. S2. ABT‐263 induces cell death in senescent LLC lung cancer cells after etoposide treatment. [file MOL2-14-2504-s002.pdf]

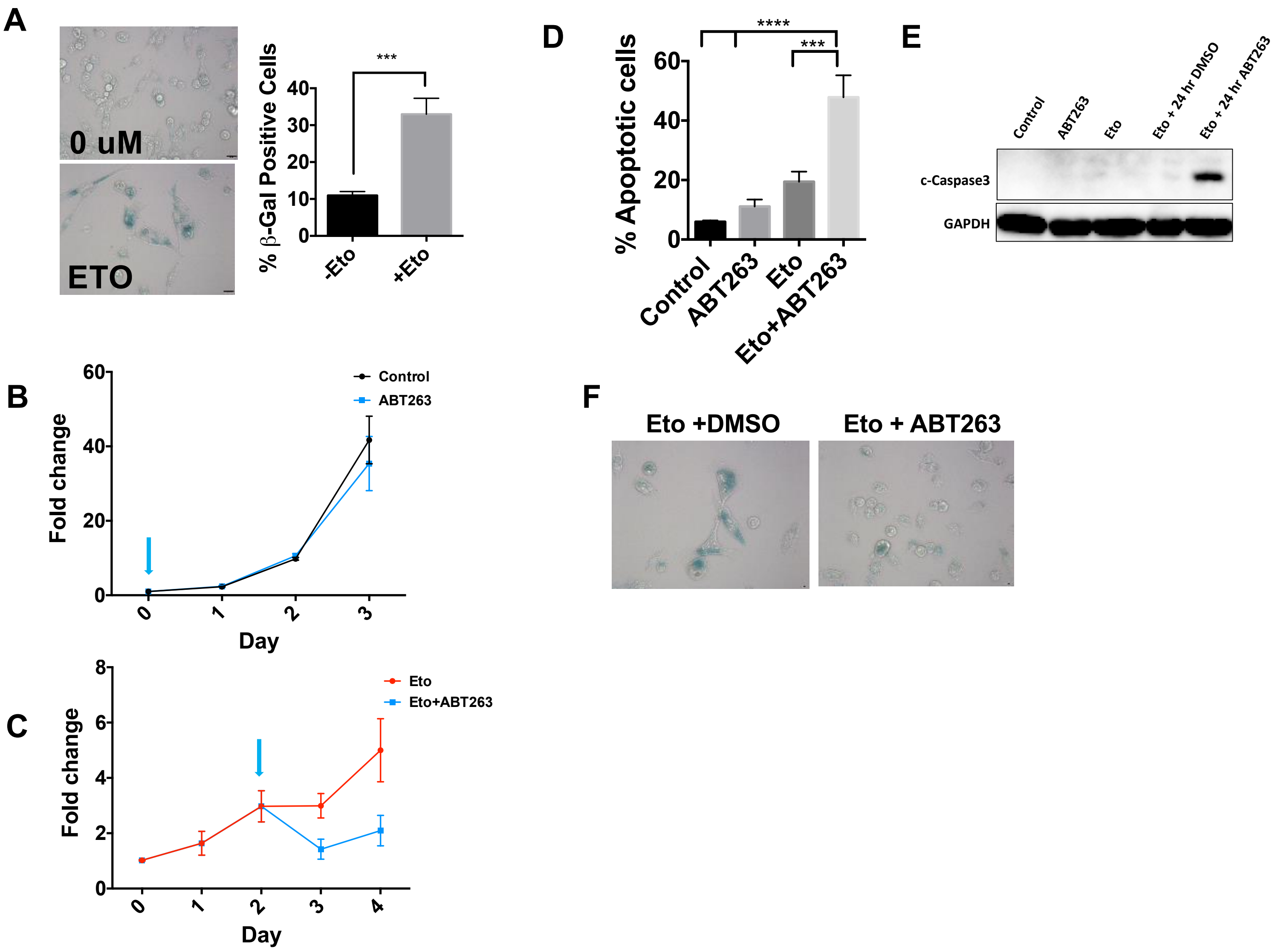
A
0 uM
ETO
% β-Gal Positive Cells
***
40
30
20
10
0
-Eto
+Eto
D
****
***
% Apoptotic cells
60
40
20
0
Control
ABT263
Eto
Eto+ABT263
E
Control
ABT263
Eto
Eto + 24 hr DMSO
Eto + 24 hr ABT263
c-Caspase3
GAPDH
B
Fold change
60
40
20
0
Control
ABT263
0
1
2
3
Day
C
Fold change
8
6
4
2
0
Eto
Eto+ABT263
0
1
2
3
4
Day
F
Eto +DMSO
Eto + ABT263

Figure. S2. ABT-263 induces cell death in senescent LLC lung cancer cells after etoposide treatment. (A) LLC cells exposed to 0.5 μM Eto for 48 hours were assessed for SA-β-gal expression by X-Gal staining and C12FDG. Eto treated cells were evaluated at the end of treatment (D2). *** $p \leq 0.001$ indicates statistical significance between untreated and Eto-treated cells as determined by an unpaired student's t-test. **(B and C)** Viable cell time courses for untreated or etoposide treated LLC cells with and without ABT-263 exposure. All conditions were exposed to 2 μM ABT-263 for 24 hours. Arrows indicate start of ABT-263 treatment. **(D)** Annexin-V/PI flow cytometry analysis for the indicated conditions. *** $p \leq 0.001$, **** $p \leq 0.0001$ indicates statistical significance between the bracketed conditions as determined by a one-way ANOVA with Tukey's post hoc test. **(E)** Western blotting for cleaved caspase three for the indicated conditions and timepoints. **(F)** X-Gal staining on Eto-treated cells after 24 hours with vehicle or ABT-263 exposure. All bright field images were captured at the same magnification (objective 20x) and are representative fields from three independent experiments (n=3). Blots are from two independent experiments (n=2), and all quantitative graphs are mean ± SEM from at three independent experiments (n=3)
