# Supplementary material for: Clearance of therapy‐induced senescent tumor cells by the senolytic ABT‐263 via interference with BCL‐XL–BAX interaction
Source: Mol Oncol. 2020 Aug 5;14(10):2504–19. doi: 10.1002/1878-0261.12761 (PMC7530780; doi:10.1002/1878-0261.12761)
Supplement: Supplementary file 3 — Fig. S3. Cells that escape ABT‐263 effects are unlike typical senescent cells. [file MOL2-14-2504-s003.pdf]

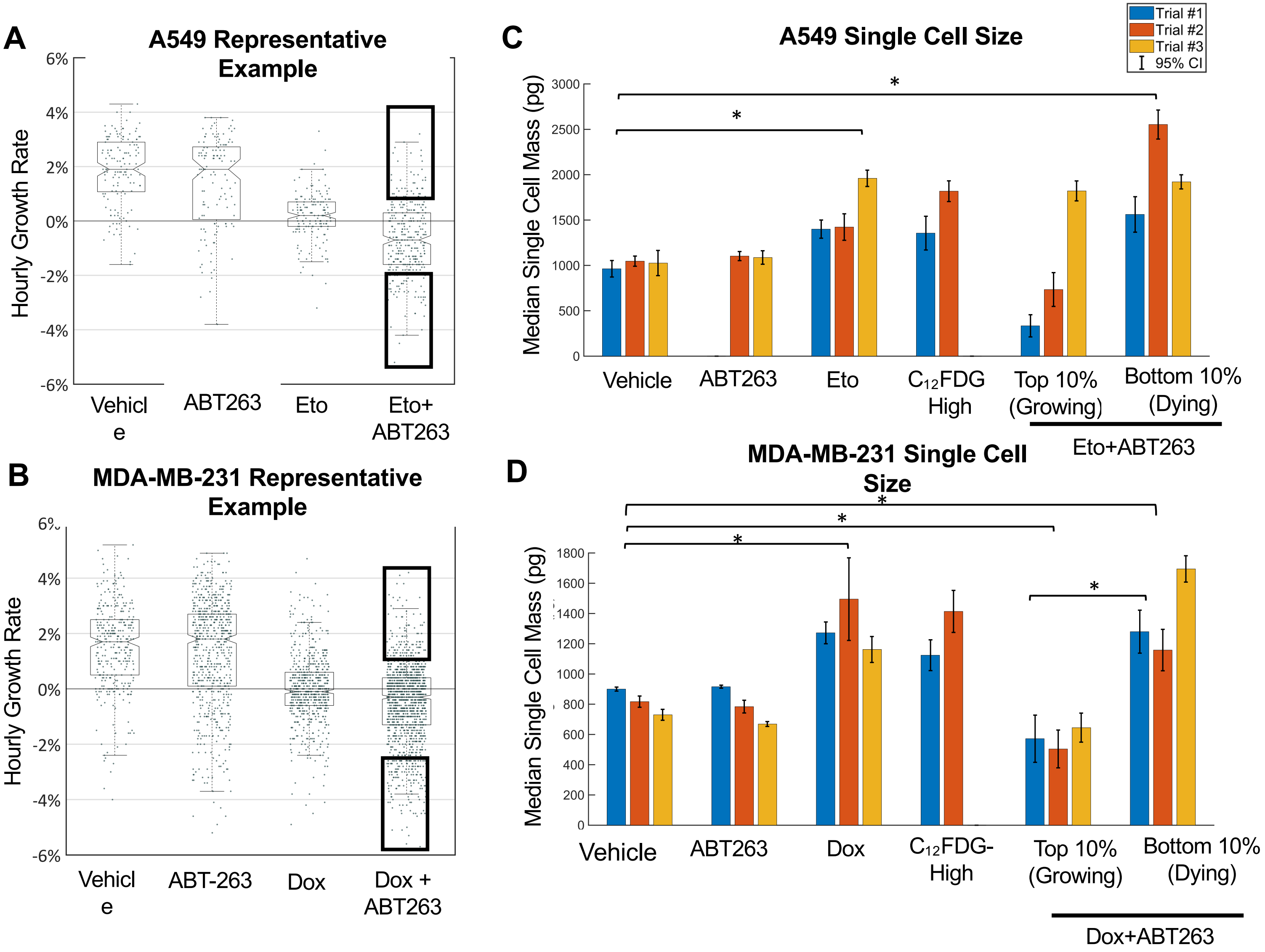
A
A549 Representative Example
Hourly Growth Rate
6%
4%
2%
0%
-2%
-4%
-6%
Vehicl e
ABT263
Eto
Eto+ ABT263
B
MDA-MB-231 Representative Example
Hourly Growth Rate
6%
4%
2%
0%
-2%
-4%
-6%
Vehicl e
ABT-263
Dox
Dox + ABT263
C
A549 Single Cell Size
Trial #1
Trial #2
Trial #3
95% CI
Median Single Cell Mass (pg)
3000
2500
2000
1500
1000
500
0
*
*
Vehicle
ABT263
Eto
C12FDG High
Top 10% (Growing)
Bottom 10% (Dying)
Eto+ABT263
D
MDA-MB-231 Single Cell Size
Median Single Cell Mass (pg)
1800
1600
1400
1200
1000
800
600
400
200
0
*
*
*
*
Vehicle
ABT263
Dox
C12FDG-High
Top 10% (Growing)
Bottom 10% (Dying)
Dox+ABT263

Figure. S3. Cells that escape ABT-263 effects are unlike typical senescent cells. (A) A549 and MDA-MB-231 cells were exposed to four conditions: vehicle, ABT-263 only, Eto or Dox, or Eto or Dox with sequential ABT-263. All conditions were monitored by HSLCI from the start of treatment with ABT-263 (time 0) to 14 hours. Individual dots in the underlying scatter plot represent the mass accumulation rates of single cells 0-14 hours post ABT-263 treatment. **(B)** MDA-MB-231 cells that were treated with vehicle, ABT-263, Dox, or Dox followed by ABT-263 were monitored by HSLCI. **(C)** The median starting mass for A549 cells that escaped ABT-263 effects (Top 10%, upper black box in A), those cells most effect by ABT-263 (Bottom 10%, lower black box in A), control, and separately treated and sorted C12FDG-high cells was determined to further characterize each group**. (D)** The median starting mass for MDA-MB-231 cells that escaped ABT-263 effects (Top 10%, upper black box in B), those cells most effect by ABT-263 (Bottom 10%, lower black box in B), control, and separately treated and sorted C12FDG-high cells were determined to further characterize each group. For both C and D, it should be noted that each experimental replicate is shown separately as previous experiments have shown variability in median cell size due to passage number. Each replicate is shown as the mean +/- SD for n=1,000 cell clusters. Each group was compared pairwise to the control group using Mood's median test. * $p \leq 0.05$
