# Supplementary material for: Clearance of therapy‐induced senescent tumor cells by the senolytic ABT‐263 via interference with BCL‐XL–BAX interaction
Source: Mol Oncol. 2020 Aug 5;14(10):2504–19. doi: 10.1002/1878-0261.12761 (PMC7530780; doi:10.1002/1878-0261.12761)
Supplement: Supplementary file 4 — Fig. S4. ABT‐199 and A‐1155463 have no effect on control cells and ABT‐263 induces apoptosis by disrupting BCL‐XL's interaction with BAX. [file MOL2-14-2504-s004.pdf]

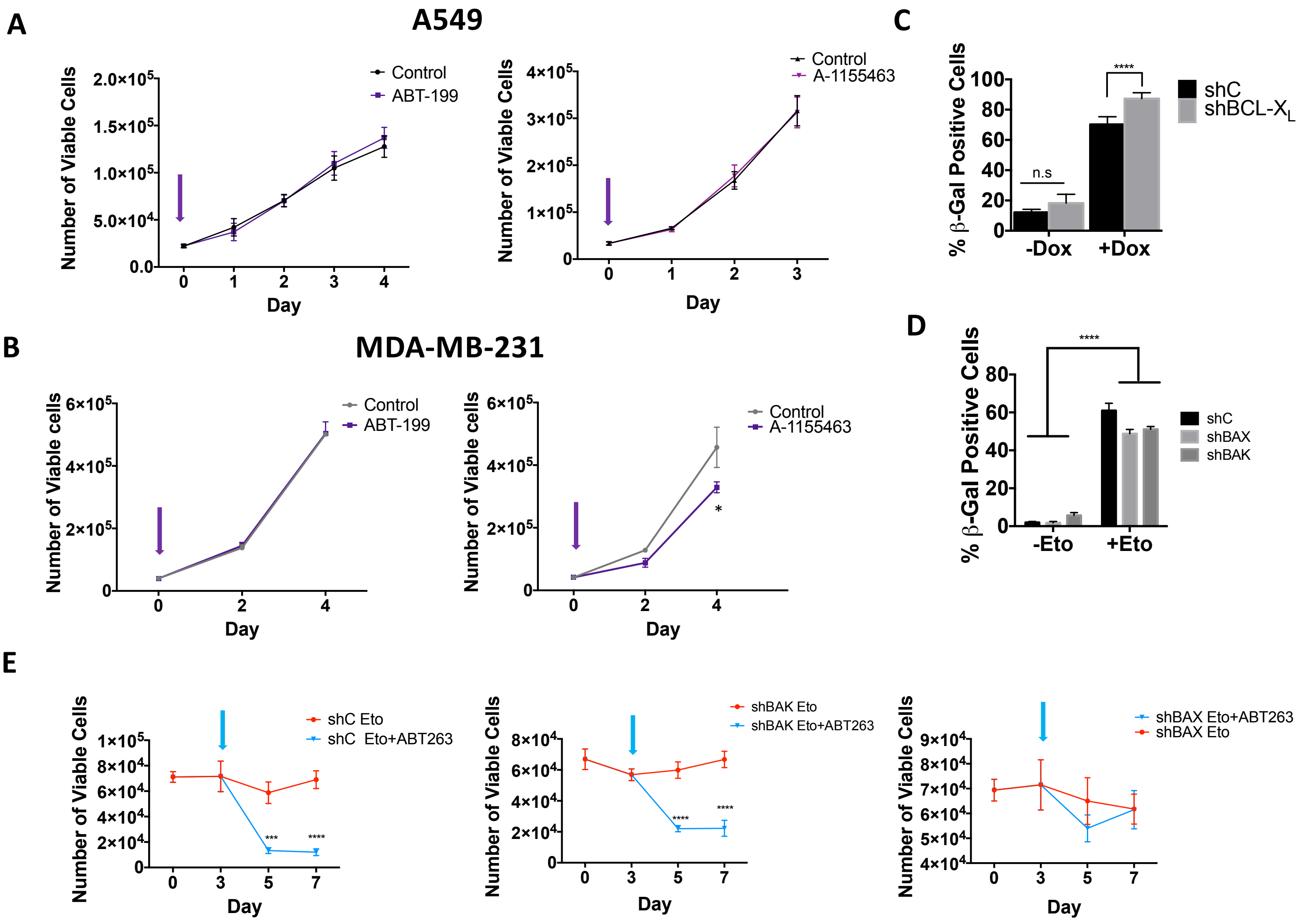

A
A549
Number of Viable Cells
2.0×10^5
1.5×10^5
1.0×10^5
5.0×10^4
0.0
Control
ABT-199
Day
4×10^5
3×10^5
2×10^5
1×10^5
Control
A-1155463
C
% β-Gal Positive Cells
shC
shBCL-XL
n.s
****
-Dox
+Dox
B
MDA-MB-231
Number of Viable cells
6×10^5
4×10^5
2×10^5
Control
ABT-199
Control
A-1155463
*
D
shC
shBAX
shBAK
-Eto
+Eto
E
shC Eto
shC Eto+ABT263
1×10^5
8×10^4
6×10^4
4×10^4
2×10^4
***
****
shBAK Eto
shBAK Eto+ABT263
shBAX Eto+ABT263
shBAX Eto
9×10^4
7×10^4
5×10^4

Figure. S4. ABT-199 and A-1155463 have no effect on control cells and ABT-263 induces apoptosis by disrupting BCL-XL's interaction with BAX. (A and B) Viable cell number for A549 cells (A) and MDA-MB-231 cells (B) treated with 2 μM ABT-199 and 2 μM A-1155463. * $p \leq 0.05$ indicates statistical significance between Eto/Dox vs. Eto/Dox + ABT-199/A-1155463 as determined by two-way ANOVA with Tukey's post hoc test. All other time-points were found to be nonsignificant. **(C)** C12FDG flow cytometry for MDA-MB-231 shBCL-X_L cells following exposure to Dox at peak senescence, Day 4. **** $p \leq 0.0001$ indicates statistical significance between shC variants and shBCL-X_L variants as determined by two-way ANOVA with Tukey's post hoc test. **(D)** C12FDG flow cytometry for A549 shC, shBAX, and shBAK cells following exposure to Eto. **** $p \leq 0.0001$ indicates statistical significance between untreated and Eto-treated cells as determined by two-way ANOVA with Tukey's post hoc test. **(E)** Viable cell number for shC, shBAK, and shBAX cells following treatment with Eto and Eto + ABT-263. *** $p \leq 0.001$, **** $p \leq 0.0001$ indicate statistical significance between Eto-treated and Eto+ABT-263 treated cells as determined by two-way ANOVA with Tukey's post hoc test. All graphs are mean ± SEM from three independent experiments (n=3)
